# Genome diversity and evolutionary characteristics of clinical isolates of *Bordetella pertussis* circulating in Iran

**Published:** 2020-02

**Authors:** Samaneh Saedi, Azadeh Safarchi, Mojtaba Noofeli, Keyvan Tadayon, Alfred Chin Yen Tay, Binit Lamichhane, Hamzeh Rahimi, Fereshteh Shahcheraghi

**Affiliations:** 1 Department of Bacteriology, Pasteur Institute of Iran, Tehran, Iran; 2 Department of Human Bacterial Vaccine, Razi Vaccine & Serum Research Institute, Agricultural Research, Education and Extension Organization (AREEO), Karaj, Iran; 3 Department of Aerobic Bacterial Research and Vaccine Production, Razi Vaccine & Serum Research Institute, Karaj, Iran; 4 The Marshall Centre for Infectious Diseases Research and Training, The University of Western Australia, Nedlands, Western Australia, Australia; 5 Shenzhen Dapeng New District Kuichong People Hospital, Shenzhen, Guangdong, China; 6 Department of Molecular Medicine, Pasteur Institute of Iran, Tehran, Iran

**Keywords:** *Bordetella pertussis*, Genome diversity, Pulsed-field gel electrophoresis, Whole genome sequencing

## Abstract

**Background and Objectives:**

The re-emergence of pertussis still is being reported all over the world. Pathogen adaptation and antigenic divergence of circulating isolates from vaccine strains are the main reasons of infection resurgence. Waning immunity is also an important factor contributing to resurgence of pertussis.

**Materials and Methods:**

The genetic diversity and evolutionary characteristics of circulating Iranian isolates of *Bordetella pertussis* during February 2015 to October 2018 was investigated by pulsed-field gel electrophoresis (PFGE) and subsequently *ptxA, ptxP* and *fim3* alleles were characterized. The next generation genome sequencing was then used to compare the genomics of *ptxP1* and *ptxP3* of selected isolates from PFGE dendrogram.

**Results:**

PFGE differentiated 62 clinical isolates and vaccine and reference strains into 19 PFGE profiles, indicating the higher level of heterogeneity in the population during 2015–2018. The predominant *B. pertussis* genotype harbored pertussis toxin promoter allele, *ptxP3* and the expansion of *ptxA1* isolates, were also observed in our population.

**Conclusion:**

No changes in allelic profile of predominant clone in recent years was observed but antigenic divergence between recently circulating isolates and the vaccine strain has been progressed and significantly was higher than previous studies. The comparative genomic analysis of the *ptxP3* and *ptxP1* isolates indicate that changes in *ptxP3* genome structure including 32 unique SNPs and three unique indels may have contributed to the expansion of the *ptxP3* clone. We compared *ptxP3* and *ptxP1* isolates in pathogenicity-associated genes and found five of them were specific for the *ptxP3* isolates. The polymorphisms in pathogenicity-associated genes suggest structural adaptations for these virulence factors.

## INTRODUCTION

Whooping cough (pertussis) is a highly contagious, acute respiratory illness of humans that is caused by the Gram-negative bacterium *Bordetella pertussis*. The infection can be life threatening in infants, who are too young to be vaccinated or are not yet fully vaccinated (1).

Introduction of whole cell vaccine (WCV) during the 1950s significantly reduced the morbidity and mortality of pertussis. However, due to the side effect of WCV, acellular vaccine (ACV) was developed in the 1980s (2). Although pertussis is relatively well controlled by extensive vaccination programs, re-emergence of pertussis has been reported in many countries with high vaccination coverage, including the European countries, the United States and Australia (3–6). Pathogen adaptation and antigenic divergence of circulating isolates from vaccine strains are the main reasons of infection resurgence. Waning immunity is also an important factor contributing to resurgence of pertussis (7, 8).

While a vast majority of developed countries switched from WCV to ACV, whole cell-based combination vaccines is still in use in developing countries (9). In Iran, the whole-cell pertussis vaccine was introduced for children in 1950s and continued until now. Children are immunized with three doses of diphtheria–tetanus, whole-cell pertussis (DTwP) vaccine at 2, 4 and 6 month. Then, young children receive two booster to maintain that protection through in 18 month and 6-years old (10). Despite high pertussis vaccination coverage in Iran, (96% since 2000) pertussis incidence is still the highest amongst all vaccine-preventable diseases. Our population has experienced pertussis resurgence since 2007 and reached its peak in 2012 and 2013 (11).

Recently, antigenic divergence has been reported in different countries. Molecular studies are important for monitoring the spread of antigenic divergence between clinical isolates and vaccine strain (12). *B. pertussis* has conventionally typed by pulsed-field gel electrophoresis (PFGE). This method has been frequently used worldwide to provide laboratory data for characterization of *B. pertussis* isolates (13). PFGE achieves some level of resolution that resulted in the high structural dynamics of *B. pertussis* genome (14, 15). On the other hand, antigenic divergence of *B. pertussis* can be easily achieved by point mutation. Genotyping of pathogenicity-associated genes is very precious to find the polymorphism of these genes. Comparative analysis of *B. pertussis* genomes by whole genome sequencing is important to study *B. pertussis* population diversity (16, 17).

The aims of this study, was to investigate the genetic diversity of recent circulating clinical *B. pertussis* isolates, using PFGE and genotyping of pathogenicity-associated genes during February 2015 to October 2018. Finally, we selected three *ptxP1* isolates and three *ptxP3* isolates from PFGE dendrogram for comparative genomics of *ptxP1* and *ptxP3* isolates, to know the cause of the *ptxP3* isolates outbreak in our population using next generation genome sequencing.

## MATERIALS AND METHODS

**Bacterial isolates.** In the current study, 62 clinical isolates were obtained from Pertussis Reference Laboratory of Pasteur Institute of Iran during February 2015 to October 2018. Isolates collected from different province of Iran including Tehran, Esfahan, Khorasan, East and West Azarbaijan, Khuzestan. Bp134, Bp509 as vaccine isolates and Bp18323 (ATCC 9797), Tohama I as reference strains, were used for validation.

Bacterial isolates were re-cultured on Regan-Lowe medium containing charcoal agar and 10% defibrinated sheep blood, and incubated at 37 °C for 72 h. *B. pertussis* isolates were confirmed by a combination of colony morphology, growth rate, Gram stain and conventional biochemical tests such as oxidase and use of specific *Bordetella* antiserums (Difco *B. pertussis* Antiserum, Rabbit serum for slide agglutination). DNA extraction was performed using High Pure PCR Template Preparation Kit (Roche Diagnostics GmbH, Mannheim, Germany). Real-time PCR was performed by targeting IS481, *ptxP*, IS1001 and IS1002 for species confirmation of *B. pertussis.* Primers and probes sequences are available in Table 1 (18).

**Table 1.** Primers and probes used in real-time PCR for amplification of the *ptxP* and insertion sequences IS481, IS1001, IS1002.

**Gene**	**Primer sequences**
*ptxP*	PT1a: 5′-GCA TGC GTG CAG ATT CGT C -3′
	PT2a: 5′-CTC TGC GTT TTG ATG GTG CCT AT -3′
	PT-FAM: 6FAM-AAT CCA ACA CGG CAT GAA CGC TCC TTC--BHQ2
IS481	IS481-F 5′ ATCAAGCACCGCTTTACCC 3′
	IS481-R 5′ TTGGGAGTTCTGGTAGGTGTG 3′
	IS481-FAM : 6FAM-AATGGCAAGGCCGAACGCTTCA BHQ1
IS1001	IS1001-F 5′ CCA GAG CCG TTT GAG TTC GT 3′
	IS1001-R 5′ AAT TGC TGC AAG CCA ACC A 3′
	IS1001-CY5 : CY5-ACA TAG ACC GTC AGC AG-BHQ-3 3′
IS1002	IS1002-F 5 ′ CTA GGT CGA GCC CTT CTT GTT AAC 3′
	IS1002-R 5′ GCG GGC AAG CCA CTT GTA 3′
	IS1002-FAM : 6FAM-CTA CGT CCA GTT CTG TTG CAT CAC CC-BHQ

**Genotypic analysis.** The polymorphism of pathogenicity-associated genes including *ptxA, ptxP* and *fim3* was determined by DNA sequencing. PCR was performed with specific primers as described previously (19–22). PCR products were purified and sent for Sanger sequencing (Macrogen, South Korea). The sequences were read by using MEGA5 software in conjunction with reference strains Tohama I (23).

**Pulsed-field gel electrophoresis.** PFGE profiles were obtained using the XbaI enzyme (Fermentas, ABI, and Germany). The restriction fragments were separated using a CHEF-DRIII system (Bio-Rad), with initial switch time: 5s, final switch: 45 s and run time: 21 h. Fragments patterns were analyzed with Gel Compare II software version 4 (Applied Maths, Belgium). *Salmonella enterica* serotype Braenderup strain H9812 was used as size marker. Dendrogram was drawn by unweighted-pair group method using the arithmetic average algorithm (UPGMA) with 2% band tolerance and 2% optimization settings with the Dice coefficient.

**Bacterial selection and whole genome sequencing.** Six isolates were selected based on the hospitalized patients, mortality and the results of PFGE dendrogram in present and our previous study (24). We choose three *ptxP1* isolates and three *ptxP3* isolates for comparative genomics analysis. Details of clinical isolates are shown in Table 3.

**Table 2.** Characteristics of pertussis patients

**Characteristic**	**Total patients N**	**(%)**
Sex distribution		
Female	35	56%
Male	27	44%
Age		
< 3 month	35	56%
> 3 month	27	44%

**Table 3.** Characteristics of *B. pertussis* isolates selected for WGS

**Sample ID**	**Age of children**	**Vaccine status**	**Hospitalized**	**PFGE Clade**	**PFGE Ref**	**Year**	***ptxA***	***ptxp***	***prn***	***Fim2***	***Fim3***	**Serotyping Fim2/*fim3***	**# Contigs**	**GC%**
IR44	1.5 Month	_	hospitalized patient	B	(24)	2012	*PtxA1*	*Ptxp1*	*Prn1*	*Fim2-1*	*Fim3 A (3–4)*	+/+	277	67.47%
IR92	2 Month	_	hospitalized patient	A (vaccine 134 cluster)	(24)	2014	*PtxA1*	*Ptxp1*	*Prn1*	*Fim2-1*	*Fim3 A (3–4)*	+/+	299	67.46%
IR133	3 Year	+	hospitalized patient	J (vaccine 134 cluster)	This study	2015	*PtxA2*	*Ptxp1*	*Prn1*	*_*	*Fim3-1*	−/+	275	67.45%
IR37	4 Month	+	hospitalized	H (Dominant cluster)	(24)	2012	*PtxA1*	*Ptxp3*	*Prn2*	*Fim2-1*	*Fim3-2*	+/+	276	67.49%
IR175	5 Year	+	hospitalized patient	A (Dominant cluster)	This study	2017	*PtxA1*	*Ptxp3*	*Prn9*	*Fim2-1*	*Fim3-2*	+/+	285	67.76%
IR178	28 Days	_	patients died	A (Dominant cluster)	This study	2018	*PtxA1*	*Ptxp3*	*Prn2*	*Fim2-1*	*Fim3-2*	−/+	282	67.91%

Genomic DNA was extracted and purified from pure culture using phenol-chloroform method (25). DNA libraries were constructed using Nextera XT kit (Illumina, San Diego, USA) according to manufacturer’s protocol and sequenced on the Illumina NextSeq instrument using 2 × 150 bp paired-end protocol. The contigs were aligned to the reference *B. pertussis* strain Tohama I (Gene Bank accession number BX470248) using progressive Mauve (version 2.3.1) and the de novo assembly of the raw reads were performed using SPAdes version 3.13.0 (26, 27). To extract Single nucleotide polymorphism (SNPs) a combination of Burrows-Wheeler Alignment (BWA) tools (version 0.7.5), Samtools (version 0.1.19) and progressive Mauve (28, 29) were used. Briefly, the filtered SNPs from mapping were compared to the SNPs exported by progressive Mauve and final SNPs were selected for further analysis. Distribution of short insertions/deletions (indels), which are less than 100 bp, were also identified using SAMtools. The nucleotide sequences have been deposited under bio sample accession number PRJNA600651. The version described in this paper is the first version.

## RESULTS

**Clinical samples characterization.** In this research, a total of 62 currently circulating Iranian *B. pertussis* isolates were selected based on year and state of isolation focusing on the states with more isolates including Tehran, Azarbaijan, Khorasan and Mazandaran. Around half of the isolates were collected from infants younger than 3 months of age 56% (n=35). The general characteristics of *Bordetella pertussis* cases are shown in Table 2. Twenty-five (40%) of the children (under 3 month) were unvaccinated and 34 (55%) were vaccinated. The results of the real time PCR showed the presence of *ptxP*, IS481, IS1002 among pertussis isolates. IS1001 was not detected among 62 clinical isolates.

**Fig. 1.**
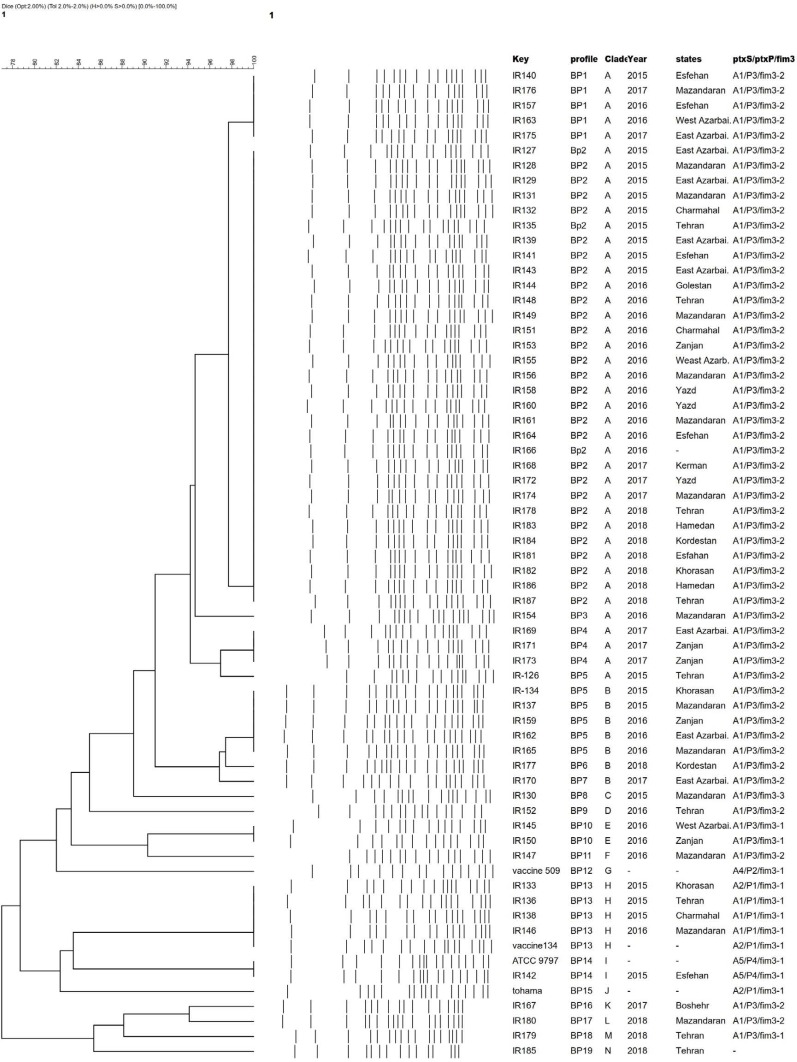
Classification of the 62 Iranian pertussis isolates, including the two reference and two vaccine strains. Dendrogram analysis of 19 PFGE profiles of *B. pertussis* isolates circulating in Iran during 2015–2018. The unweighted pair group method using arithmetic averages (UPGMA) with 2% band tolerance, 2% optimization settings and cut off 94% was used as the clustering method.

**Genotypic analysis.** The pertussis-toxin subunit A *(ptxA)*, *fim3* and pertussis toxin promoter *(ptxP)* were genotyped. Four *ptxA (ptxA1, ptxA2* and *ptxA4, ptxA5)*, four *ptxP (ptxP1, ptxP2, ptxP3* and *ptxP4)* three *fim3 (fim3-1, fim3-2, fim3-3)* alleles were detected among clinical isolates, vaccine isolates and the reference genomes (Bp134, Bp509, Bp18323, Tohama I). The frequencies of the alleles were analyzed in our phylogenetic tree (Fig. 1). Results revealed that *ptxP3* allele were predominant among local isolates with frequencies of 90.3% (n=56) and it has been a significant expansion compared to years before 2015. *ptxA1* allele was predominant with frequencies of 96.6% (n=60). Data on *fim3* sequencing showed that, the predominant allele is *fim3-2* with frequencies of 83.8% among clinical and vaccine isolates. Only one isolate had *ptxA2* (IR133) meaning that there is an allelic mismatch between the currently circulating strain and the vaccine strain. Out of the 56 *ptxP3* isolates, 52 isolates carried *fim3-2*. Four isolates were *ptxP1* which were further differentiated by *ptxA* allele into *ptxP1/ptxA1/fim3-1* (IR136, IR138, IR146) and *ptxP1/ptxA2/fim3-1* (one isolate). *ptxA1/ptxP3/ fim3-2* were determined as the predominant allelic profile circulating in Iran (85%) while vaccine seed strain Bp134 showed *ptxA2/ptxP1/fim3-1* and vaccine seed isolates Bp509 showed *ptxA4/ptxP2/fim3-1* allelic profile.

**PFGE analysis of Iranian *B. pertussis* isolates from 2015 to 2018**. PFGE was highly discriminatory between our isolates, and identified 19 distinct PFGE profiles, named BP_1_ – BP_19_ (Fig. 1). All profiles were closely related to each other and based on similarity higher than 95%, they were classified into 14 major clades (A–N). Four profiles consisted of closely related isolates (BP_1_ - BP_4_). The major profile was clade A, which consisted of 41 isolates and characterized by *ptxA1/ptxP3/fim3-2*. BP2 as a major group among isolates, comprised 31 isolates that classified in clade A.

Clade H indicated the same profile as the Iranian vaccine seed strain Bp134 with a little diversity in *ptxA* antigen (due to point mutation). In clade I, only one isolate, grouped with reference strain BP18323 (9797) with the same PFGE pattern. This strain (strain no. IR142) was isolated in 2015 with the similar allelic profile to *B. pertussis* 18323 *(ptxA5/ptxP4/ fim3-1)* and had low similarity with all clinical isolates tested. Finally, we found no correlation between year and state of isolation in PFGE dendrogram.

**Whole genome sequencing and identification of single nucleotide polymorphisms.** In this study, we illustrated comparative genomics of *ptxP1* and *ptxP3* isolates (Table 3). We calculated an approximate genome size for each isolate using qualimap. The average genome size of our clinical isolates is 3, 885, 151 bp. A total of 366 SNPs was identified, of which 102 SNPs were in intergenic (IG) region and 264 located in genes, while 149 SNPs were non-synonymous (nsSNPs), 115 SNPs were synonymous (sSNPs).

Twenty SNPs were common in all isolates, among them, eight were intergenic (IG) and 15 located in genes. The nsSNPs located in BP1660 *(sphB2)*, found in all isolates, is an auto transporter and involved in pertussis pathogenicity. *PtxP3* isolates differentiated from *ptxP1* isolates by 32 unique SNPs, among them seven were intergenic and 24 SNPs were in coding regions (Table 4). There is one *ptxP3* isolate (IR175), carrying *prn9* allele. Thirteen unique SNPs were detected related to *prn9* isolate with *ptxA1/ptxp3/ fim3-2/fim2-1* antigenic profile. The main nsSNPs mutations located in BP1226 (conserved hypothetical), BP2548 (regulation) and BP3405, nsSNPs in BP3405 (ompQ) were only in *prn9* Iranian isolates.

**Table 4.** List of unique SNPs in *PTXP3* isolates

**Site**	**TYPE**	**Mutation**	**GENE_ID**	**Site**	**TYPE**	**Mutation**		**GENE_ID**
36857	intergenic	A	G		1290405	sSNP	G	A	BP1227
50044	intergenic	C	G		1331840	intergenic	G	A	
115891	nsSNP	T	A	BP0118	1134238	nsSNP	C	A	BP1086
185405	sSNP	G	A	BP0184	1547488	nsSNP	A	G	BP1471
196307	nsSNP	T	C	BP0194	1647861	nsSNP	C	A	BP1568
220937	sSNP	G	A	BP0215	1827556	intergenic	G	A	
299559	intergenic	C	T		2374322	nsSNP	T	C	BP2249
511992	intergenic	A	G		2505238	intergenic	T	C	
514171	intergenic	G	A		2651008	nsSNP	G	A	BP2502
517207	sSNP	G	T	BP0505	3263622	intergenic	A	C	
518837	sSNP	T	C	BP0507	3591185	intergenic	T	C	
525420	nsSNP	G	C	BP0518	3840411	nsSNP	G	A	BP3630
654224	nsSNP	G	A	BP0646	3988168	intergenic	G	A	
694521	sSNP	A	G	BP0678	3991376	sSNP	C	T	BP3787
883816	nsSNP	C	T	BP0854	4068047	nsSNP	C	T	BP3857
1098918	sSNP	T	C	BP1054	4068650	nsSNP	C	T	BP3858
1098922	nsSNP	G	T	BP1054	4071996	sSNP	G	A	BP3861

**Fig. 2.**
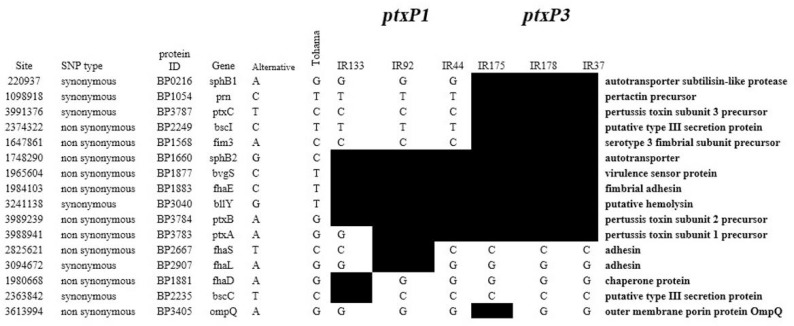
Polymorphisms found in pathogenicity-associated genes in *ptxP1* (IR133, IR92, IR44) and *ptxP3* (IR175, IR178, IR37) isolates. The distribution of polymorphisms are distinguished by black color.

We compared *ptxP3* and *ptxP1* isolates in pathogenicity-associated genes, we found five of them were specific for the *ptxP3* isolates (Fig. 2). Polymorphisms were found in pathogenicity-associated genes suggesting structural adaptations for these virulence factors.

**Fig. 3.**
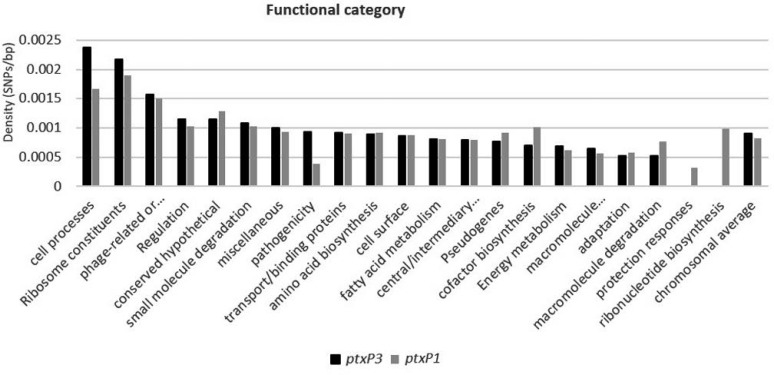
Comparative SNP densities between *ptxP3* and *ptxP1* strains based on functional category.

On the other hand, the SNP density (the number of SNPs per bp of gene in each category) of *ptxP3* and *ptxP1* isolates was calculated. The overall SNP density of the whole genomes were, 0.0008 SNPs/bp with uses of functional categories defined by Parkhill et al. (30). The highest SNP density were in ribosome constituents (0.002SNPs/bp), phage-related or transposon-related (0.0014SNPs/bp) and regulation (0.0011SNPs/bp). The main SNP density differences between *ptxP3* and *ptxP1* isolates in cell processes and ribosome constituents that shows in Fig. 3.

Finally, a total of 40 indels were found in our isolates, 24 deletion and 16 insertion. Twenty-four indels were in genes and 16 were intergenic (IG). Among indels located in genes, 19 were frameshifts, of which 10 resulted from single base pair (bp) indels, two from three bp indels and six each from 4, 7, 8 and 31 bp indels. Two frameshifts indels located in BP2232, BP2928 had a stop codon resulting in proteins that were shorter than expected which were considered as pseudogenes (30). Three indels were unique in *ptxP3*, all as frameshift mutations. BP0880 was a pseudogene encoding for a putative exported protein and BP1054 codes *prn* precursor that cause *prn* new allele production (Table 5).

**Table 5.** List of Indels in *PTXP3* isolates

**Gene ID**	**Position**	**Name**	**Function**	**Deletion/Insertion**
BP0880	919013		Putative exported protein	Deletion (frameshift mutation occurs)
BP1054	1098926	prn	Prn precursor	Insertion
BP3224	3436723		Putative cytochrome oxidase	Insertion

## DISCUSSION

When the first pertussis surveillance system has started monitoring whooping cough in our country, predominant profile expresses *ptxP3/ptxA1/fim3-2*. In previous study carried out by our team analyzed the alle typing and PFGE profile of clinical *B. pertussis* isolates collected during 2008– 2015. In this study we continued PFGE typing of new isolates collected during 2015–2018. In previous study, Heravi et al. showed that the majority of current circulating *B. pertussis* isolates in Iran are *ptxP3* strains following the worldwide pattern (24). In the current study, there was no changes in allelic profile of predominant clone in recent years but antigenic divergence between recently circulating isolates and the vaccine strain has progressed and significantly is higher than previous years. We found that almost all circulating isolates harbored *ptxP3, ptxA1* and *fim3-2* alleles, while our vaccine seed isolates Bp134 shows *ptxA2/ ptxP1/fim3-1* alleleic profile and vaccine seed isolates Bp509 shows *ptxA4/ptxP2/fim3-1*. Divergences between the vaccine isolates and PFGE predominant clade are clear and may have effect on vaccine efficacy.

In the present study, we used PFGE, as a tool to monitor the pertussis population. Nineteen distinct PFGE profiles from phylogenetic analysis of Iranian pertussis population indicated the higher level of heterogeneity in the population during 2015–2018. Some PFGE patterns of currently predominant *B. pertussis* clones were found within PFGE groups BP2 that classified in clade A, this predominant clonal type represent a single profile identical to the European profile BpSR11, which is part of PFGE group IV (31, 32). This profile comprised 10 to 50% of the isolates, from all European countries (except Poland) and characterized to possess *ptxA1/ptxP3/fim3-2* (32–34). Similar PFGE profiles were observed in Canada with 82% of the isolates represented, *ptxA1/ptxP3/fim3-2* and belonged to PFGE group IV (35). In these countries vaccinations with WCV were started 50 to 60 years ago and switched to ACV 10 to 20 couple of years ago (32, 35, 36). Iranian predominant profiles are different from other countries using whole cell vaccines such as Poland. The replacement of the *B. pertussis* population in Iran by Clade A with *ptxP3/ ptxA1* profile, where only WCV has been used, suggests that the expansion of *ptxP3* strains was not driven by ACV selection alone. In Poland where WCV has also been in use for decades, in 2000–2013, *ptxP1* isolates predominated with 77.5%. However, in a recent study in Poland during 2010–2016, 61.5% of the isolates were found to be *ptxP3* and were attributed to the increasing use of ACV since 2013 for primary immunization. (37, 38). We conclude that, the rate of predominant allelic profiles not necessarily related to the type of population vaccine.

In the last ten years two *ptxP* alleles, *ptxP1* and *ptxP3*, were predominated among the Iranian populations of *B. pertussis*. The *ptxP3* isolates were observed in 2005 for the first time and then gradually increased in frequency and now in recent years has been replaced by *ptxP1* isolates, while we had only 16.7% *ptxP1* among our isolates. Predominant *ptxP3* profile appears to have infection-associated biologic characteristics such as higher *Ptx* production and enhanced respiratory colonization which contributed to the pertussis epidemic that differ from those of *ptxP1* isolates (21, 39, 40).

Recently, whole-genome sequencing has been used to identify genetic changes in the *B. pertussis* population. For better understanding of *ptxP3* and *ptxP1* genomic diversity, we compared whole genome sequences of *ptxP3* genomes and *ptxP1* isolates. We confirmed that *ptxP3* isolates differed from *ptxP1* isolates in genomics. There are 32 unique SNPs that differentiated the *ptxP3* isolates from *ptxP1* isolates. The most important mutations in the virulence genes unique for *ptxP3* strains that occurs in *prn, ptxC* and *fim3*, responsible for changes in alleles of the genes encoding vaccine antigens and may be the cause of *ptxP3* lineage expansion in our population. Our results showed that *ptxP3* genomic profile is similar to those collected worldwild due to the better genome fitness of *ptxP3* isolates, the most important mutations in the virulence genes unique for *ptxP3* isolates that occurs in *sphB1, prn, ptxC, bscI* and *fim3*, responsible for changes in alleles of the genes encoding vaccine antigens and may be the cause of *ptxP3* lineage expansion (40, 41). BP2249 *(bscI)*, BP1568 *(fim3)*, that maybe adaptive and due to the emergence of a new allele, nsSNPs in *fim3* was involved in antigenic shift from *fim3-1* to *fim3-2* (5).

On the other hand, *ptxP1*, contains isolates which represent *ptxA1/ptxP1* and *ptxA2/ptxP1. PtxA1/ptxP1* was distinguished by 35 unique SNPs from *ptxA2/ ptxP1* isolates. The main non-synonymous mutations found in *ptxP1/ptxA1* profile located in BP3812 and BP3869 are involved in cell surface, BP1568 encoding *fim3* involve in pathogenicity, and BP3137, putative two-component system sensor protein involved in regulation.

In conclusion, currently in Iran the surveillance system is affected by many limitations. The underestimation of pertussis in adolescents and adults is mainly related to the atypical clinical characteristics of cases and lack of lab confirmation, the age group is considered as the reservoir of infection.

Epidemiological results of sequencing and PFGE is very helpful to control and prevention of pertussis. However, Iran has not experienced pertussis epidemic after 2013, but our finding suggest the fact that the presence of non-vaccine *ptxP3/ptxA1*, may associate with rising trend of pertussis among populations since 2007. The comparative genomic analysis of the *ptxP3* and *ptxP1* isolates provided new insights into the evolution of pertussis isolates in Iran. Our findings illustrate that changes in *ptxP3* genome structure including 32 unique SNPs and three unique indels may have contributed to the expansion of the *ptxP3* clone. Furthermore, this is the first study about comparative genome analysis of *ptxP1* and *ptxP3* allele type with next generation sequencing, in Iran. These results could be impressive in controlling of infection and help policy makers in the healthcare and community settings but there is a need to perform more sequence of *ptxP3* isolates in order to obtain a better picture of the pathogen adaptation under vaccine pressure.

## References

[1] Clark TA. Changing pertussis epidemiology: everything old is new again. J Infect Dis 2014;209:978–981. 24626532 10.1093/infdis/jiu001

[2] Advani A Hallander HO Dalby T Krogfelt KA Guiso N Njamkepo E Pulsed-field gel electrophoresis analysis of *Bordetella pertussis* isolates circulating in Europe from 1998 to 2009. J Clin Microbiol 2013;51:422–428. 23175253 10.1128/JCM.02036-12 PMC3553888

[3] Winter K Glaser C Watt J Harriman K Centers for Disease Control and Prevention (CDC). Pertussis epidemic California, 2014. MMWR Morb Mortal Wkly Rep 2014;63:1129–1132. 25474033 PMC4584602

[4] Safarchi A Octavia S Wu SZ Kaur S Sintchenko V Gilbert GL Genomic dissection of Australian *Bordetella pertussis* isolates from the 2008–2012 epidemic. J Infect 2016;72:468–477. 26826518 10.1016/j.jinf.2016.01.005

[5] Sealey KL Harris SR Fry NK Hurst LD Gorringe AR Parkhill J Genomic analysis of isolates from the United Kingdom 2012 pertussis outbreak reveals that vaccine antigen genes are unusually fast evolving. J Infect Dis 2015;212:294–301. 25489002 10.1093/infdis/jiu665

[6] Cherry JD. Epidemic pertussis in 2012—the resurgence of a vaccine-preventable disease. N Engl J Med 2012;367:785–787. 22894554 10.1056/NEJMp1209051

[7] Bart MJ Harris SR Advani A Arakawa Y Bottero D Bouchez V Global population structure and evolution of *Bordetella pertussis* and their relationship with vaccination. mBio 2014;5(2):e01074. 24757216 10.1128/mBio.01074-14 PMC3994516

[8] Bahmanjeh A Noofeli M Khaki P Hassanzadeh SM. Genetic analysis of clinical and vaccine strains of *Bordetella pertussis* by pulsed-field gel electrophoresis (PFGE), multi locus sequence typing (MLST) and serotyping. Comp Immunol Microbiol Infect Dis 2019;64:168–175. 31174694 10.1016/j.cimid.2019.03.010

[9] Dias W van der Ark AA Sakauchi MA Kubrusly FS Prestes AFR Borges MM An improved whole cell pertussis vaccine with reduced content of endotoxin. Hum Vaccin Immunother 2013;9:339–348. 23291935 10.4161/hv.22847 PMC3859757

[10] Moradi-Lakeh M Esteghamati A. National immunization program in Iran: whys and why nots. Hum Vaccin Immunother 2013;9:112–114. 23442584 10.4161/hv.22521 PMC3667923

[11] World Health Organization. WHO vaccine-preventable diseases: monitoring system 2013 global summary. 2013 Immunization schedule for. 2013;6.

[12] Mooi FR. *Bordetella pertussis* and vaccination: the persistence of a genetically monomorphic pathogen. Infect Genet Evol 2010;10:36–49. 19879977 10.1016/j.meegid.2009.10.007

[13] De Moissac Y Ronald SL Peppler MS. Use of pulsed-field gel electrophoresis for epidemiological study of *Bordetella pertussis* in a whooping cough outbreak. J Clin Microbiol 1994;32:398–402. 8150949 10.1128/jcm.32.2.398-402.1994 PMC263043

[14] Bowden KE Williams MM Cassiday PK Milton A Pawloski L Harrison M Molecular epidemiology of pertussis epidemic—Washington State, 2012. J Clin Microbiol 2014;52:3549–3557. 25031439 10.1128/JCM.01189-14 PMC4187741

[15] Weigand MR Peng Y Loparev V Batra D Bowden KE Burroughs M The history of *Bordetella pertussis* genome evolution includes structural rearrangement. J Bacteriol 2017;199(8): e00806–00816. 28167525 10.1128/JB.00806-16 PMC5370423

[16] Bart MJ van Gent M van der Heide HG Boekhorst J Hermans P Parkhill J Comparative genomics of prevaccination and modern *Bordetella pertussis* strains. BMC Genomics 2010;11:627. 21070624 10.1186/1471-2164-11-627 PMC3018138

[17] Belcher T Preston A. *Bordetella pertussis* evolution in the (functional) genomics era. Pathog Dis 2015;73:ftv064. 26297914 10.1093/femspd/ftv064 PMC4626590

[18] Reischl U Lehn N Sanden GN Loeffelholz MJ. Real-time PCR assay targeting IS481 of *Bordetella pertussis* and molecular basis for detecting *Bordetella holmesii*. J Clin Microbiol 2001;39:1963–1966. 11326023 10.1128/JCM.39.5.1963-1966.2001 PMC88058

[19] Nikbin VS Ahmadi NJ Hosseinpour M Lotfi MN Shooraj F Sadeghpour F Virulence factors variation among *Bordetella pertussis* isolates in Iran. Int J Mol Cell Med 2015;4:138–142. 26261803 PMC4499576

[20] Bottero D Gaillard ME Fingermann M Weltman G Fernández J Sisti F Pulsed-field gel electrophoresis, pertactin, pertussis toxin S1 subunit polymorphisms, and surfaceome analysis of vaccine and clinical *Bordetella pertussis* strains. Clin Vaccine Immunol 2007;14:1490–1498. 17699837 10.1128/CVI.00177-07 PMC2168178

[21] Mooi FR van Loo IH Van Gent M He Q Bart MJ Heuvelman KJ *Bordetella pertussis* strains with increased toxin production associated with pertussis resurgence. Emerg Infect Dis 2009;15:1206–1213. 19751581 10.3201/eid1508.081511 PMC2815961

[22] Haghighi F Shahcheraghi F Abbasi E Eshraghi SS Zeraati H Mousavi SAJ Genetic profile variation in vaccine strains and clinical isolates of *Bordetella pertussis* recovered from Iranian patients. Avicenna J Med Biotechnol 2014;6:178–184. 25215182 PMC4147105

[23] Tamura K Peterson D Peterson N Stecher G Nei M Kumar S. MEGA5: molecular evolutionary genetics analysis using maximum likelihood, evolutionary distance, and maximum parsimony methods. Mol Biol Evol 2011;28:2731–2739. 21546353 10.1093/molbev/msr121 PMC3203626

[24] Heravi FS Nikbin VS Lotfi MN Badiri P Ahmadi NJ Zahraei SM Strain variation and antigenic divergence among *Bordetella pertussis* circulating strains isolated from patients in Iran. Eur J Clin Microbiol Infect Dis 2018;37:1893–1900. 30094521 10.1007/s10096-018-3323-6

[25] Octavia S Lan R. Frequent recombination and low level of clonality within *Salmonella enterica* subspecies I. Microbiology 2006;152:1099–1108. 16549673 10.1099/mic.0.28486-0

[26] Darling AE Mau B Perna NT. progressiveMauve: multiple genome alignment with gene gain, loss and rearrangement. PLoS One 2010;5(6):e11147. 20593022 10.1371/journal.pone.0011147 PMC2892488

[27] Bankevich A Nurk S Antipov D Gurevich AA Dvorkin M Kulikov AS SPAdes: a new genome assembly algorithm and its applications to single-cell sequencing. J Comput Biol 2012;19:455–477. 22506599 10.1089/cmb.2012.0021 PMC3342519

[28] Li H Durbin R. Fast and accurate short read alignment with Burrows–Wheeler transfor m. Bioinformatics 2009;25:1754–1760. 19451168 10.1093/bioinformatics/btp324 PMC2705234

[29] Li H Handsaker B Wysoker A Fennell T Ruan J Homer N The sequence alignment/map format and SAMtools. Bioinformatics 2009;25:2078–2079. 19505943 10.1093/bioinformatics/btp352 PMC2723002

[30] Parkhill J Sebaihia M Preston A Murphy LD Thomson N Harris DE Comparative analysis of the genome sequences of *Bordetella pertussis*, Bordetella parapertussis and Bordetella bronchiseptica. Nat Genet 2003;35:32–40. 12910271 10.1038/ng1227

[31] Advani A Donnelly D Hallander H. Reference system for characterization of *Bordetella pertussis* pulsed-field gel electrophoresis profiles. J Clin Microbiol 2004;42:2890–2897. 15243034 10.1128/JCM.42.7.2890-2897.2004 PMC446263

[32] Hallander H Advani A Riffelmann M Von König CH Caro V Guiso N *Bordetella pertussis* strains circulating in Europe in 1999 to 2004 as determined by pulsed-field gel electrophoresis. J Clin Microbiol 2007;45:3257–3562. 17699646 10.1128/JCM.00864-07 PMC2045341

[33] Barkoff A-M Mertsola J Pierard D Dalby T Hoegh SV Guillot S Surveillance of circulating *Bordetella pertussis* strains in Europe during 1998–2015. J Clin Microbiol 2018 4 25;56(5): e01998–02017. 29491017 10.1128/JCM.01998-17 PMC5925733

[34] Van Gent M Heuvelman C Van der Heide H Hallander H Advani A Guiso N Analysis of *Bordetella pertussis* clinical isolates circulating in European countries during the period 1998–2012. Eur J Clin Microbiol Infect Dis 2015;34:821–830. 25527446 10.1007/s10096-014-2297-2 PMC4365279

[35] Shuel M Jamieson FB Tang P Brown S Farrell D Martin I Genetic analysis of *Bordetella pertussis* in Ontario, Canada reveals one predominant clone. Int J Infect Dis 2013;17(6):e413–417. 23352492 10.1016/j.ijid.2012.12.015

[36] Octavia S Sintchenko V Gilbert GL Lawrence A Keil AD Hogg G Newly emerging clones of *Bordetella pertussis* carrying *prn2* and *ptxP3* alleles implicated in Australian pertussis epidemic in 2008–2010. J Infect Dis 2012;205:1220–1224. 22416243 10.1093/infdis/jis178

[37] Mosiej E Augustynowicz E Zawadka M Dąbrowski W Lutyńska A. Strain variation among *Bordetella pertussis* isolates circulating in Poland, after 50 years of whole-cell pertussis vaccine use. J Clin Microbiol 2011;49:1452–1457. 21307213 10.1128/JCM.01487-10 PMC3122874

[38] Polak M Zasada AA Mosiej E Krysztopa-Grzybowska K Witkowski L Rzeczkowska M Pertactin-deficient *Bordetella pertussis* isolates in Poland—a country with whole-cell pertussis primary vaccination. Microbes Infect 2019;21:170–175. 30580013 10.1016/j.micinf.2018.12.001

[39] de Gouw D Hermans PW Bootsma HJ Zomer A Heuvelman K Diavatopoulos DA Differentially expressed genes in *Bordetella pertussis* strains belonging to a lineage which recently spread globally. PLoS One 2014;9(1):e84523. 24416242 10.1371/journal.pone.0084523 PMC3885589

[40] King AJ van der Lee S Mohangoo A van Gent M van der Ark A van de Waterbeemd B. Genome-wide gene expression analysis of *Bordetella pertussis* isolates associated with a resurgence in pertussis: elucidation of factors involved in the increased fitness of epidemic strains. PLoS One 2013;8(6):e66150. 23776625 10.1371/journal.pone.0066150 PMC3679012

[41] Safarchi A Octavia S Luu LDW Tay CY Sintchenko V Wood N Better colonisation of newly emerged *Bordetella pertussis* in the co-infection mouse model study. Vaccine 2016;34:3967–3971. 27346304 10.1016/j.vaccine.2016.06.052

